# A trial of nonspecific immunotherapy using systemic C. parvum in treated patients with Dukes B and C colorectal cancer.

**DOI:** 10.1038/bjc.1982.86

**Published:** 1982-04

**Authors:** R. G. Souter, P. G. Gill, P. J. Morris

## Abstract

In view of the relatively poor prognosis for patients after surgery for locally invasive colorectal cancer a trial of repeated systemic infusions of Corynebacterium parvum (CP) has been carried out. It is in this group of patients, with a high risk of recurrence from small residues of cancer left by the surgeon, that immunotherapy should have its optimum chance of success. A total of 92 patients were included in a randomized controlled study. The two groups were comparable in terms of tumour stage at presentation, operation performed and mean age of patients, but the control group had a preponderance of male patients. The study was carried out over 54 months. Treatment resulted in greater side effects than had been predicted, and as a result many patients could not be considered for inclusion.


*Br. J. Cancer* (1982) **45**, 506

### A TRIAL OF NONSPECIFIC IMMUNOTHERAPY USING SYSTEMIC *C. PARVUM* IN TREATED PATIENTS WITH DUKES B AND C COLORECTAL CANCER

R. G. SOUTER, P. G. GILL* AND P. J. MORRIS

*From the John Radcliffe Hospital, Headington, Oxford*

Received 20 November 1981 Accepted 14 February 1982

**Summary.—In view of the relatively poor prognosis for patients after surgery for locally invasive colorectal cancer a trial of repeated systemic infusions of *Corynebacterium parvum* (CP) has been carried out. It is in this group of patients, with a high risk of recurrence from small residues of cancer left by the surgeon, that immunotherapy should have its optimum chance of success.**

**A total of 92 patients were included in a randomized controlled study. The two groups were comparable in terms of tumour stage at presentation, operation performed and mean age of patients, but the control group had a preponderance of male patients. The study was carried out over 54 months. Treatment resulted in greater side effects than had been predicted, and as a result many patients could not be considered for inclusion.**

IN 1977 CANCER of the colon and rectum caused 16,462 deaths in England and Wales (Office of Population Censuses and Surveys, 1977). Advances in surgical technique have not improved the prospects of a cure for the patient presenting with the disease. Surgical treatment in the Oxford region (Gill & Morris, 1978) gives results similar to those from Birmingham (Slaney, 1971) and Bristol (Walker, 1972). After excision of Dukes B cancers, patients had an age-adjusted 5-year survival rate of 58%, and for Dukes C it was 27·1%. While specialized centres of referral can report figures considerably better than these (Whittaker & Goligher 1976; Lockhart-Mummery *et al.*, 1976), it seems likely that our own results are more representative of centres where a substantial proportion of cases of colorectal cancer present as emergencies. These results are particularly depressing when it is considered how often the surgeon is confident that he has removed all traces of malignant tissue.

It is against this disappointing background that consideration has been given to additional treatment after surgical excision of these cancers (MacDonald, 1976; Carter 1976). One approach is based on a postulated role for a specific immune defence mechanism in the host with colorectal cancer, with the concept that this is depressed in these patients and might be restored to normal by appropriate immunostimulation. If a specific defence mechanism did exist in colorectal cancer (or in any cancer for that matter), tumour-specific antigens would be present and should be identifiable. Conclusive proof for this, however, is lacking.

Several *in vitro* and *in vivo* studies support the view that tumour-specific antigens are present in colorectal cancer, and that they may evoke an immune response in the tumour-bearing patient (Gold & Freedman, 1965; Nairn *et al.*, 1971; Pihl *et al.*, 1976; Vose *et al.*, 1981; Hollinshead *et al.*, 1972; Bull *et al.*, 1973; Wanebo *et al.*, 1980). Although the clinical importance of these observations is not

* Current address: The Royal Adelaide Hospital, Australia.

yet clear, it seems reasonable to assume that the restoration of immune reactivity, either specific or nonspecific, might influence the development and spread of disease.

The anaerobic diphtheroid bacterium, *Corynebacterium parvum* (CP) has been extensively investigated in the treatment of experimental animals bearing tumours (Scott 1974 *a,b*). Its effect is probably mediated by mononuclear phagocytes when the bacterium is used systemically, and CP treatment suppresses the development of many transplantable tumours (Scott, 1974*a*). Early reports of the use of CP in humans suffering from advanced cancers suggested that the treatment was well tolerated and led to prolonged remission of disease in some patients (Israël, 1975).

In view of the poor prognosis for patients who present with Dukes B and C colorectal cancers, this group was selected for a trial of regular systemic infusions of CP over the 2 years after surgical excision of their tumours. Many relapses occur in this period, and immune stimulation might be expected to be of maximal benefit when used in a patient bearing minimal residual disease (Carter, 1976). It was also of interest to see whether CP treatment would influence the development of distant metastases, since animal experiments suggested that systemic use of the bacterium would have a greater effect in suppressing metastases than local recurrence of tumour (Sadler & Castro, 1976).

### PATIENTS AND MATERIALS

Patients were considered for inclusion in the trial during convalescence after surgery for pathologically confirmed Dukes B and C carcinoma of the colon or rectum. Dukes A patients were excluded because of their excellent prognosis. Patients with advanced disease and distant metastases were excluded because of the improbability of immune stimulation being effective against a large bulk of residual disease.

Before randomization the side effects and nature of the treatment were explained to all patients considered for inclusion in the trial.

Treatment involved a series of i.v. infusions of CP in a dose of 5 mg/m$^2$ given in a diluting volume of 100 ml of normal saline over a period of 30 min. The first treatment was within a month of operation, and in each patient it was planned to give 10 infusions in the 2 years after surgery. The treatment interval was monthly for the first 3 infusions, 2-monthly for the next 3, and then in the second year after surgery the last 4 were given 3-monthly. Treatment usually involved an overnight stay in hospital, but some patients were allowed home within 8 h of the infusion. In our preliminary pilot study, and from other reports, it was evident that the side-effects of i.v. CP included hypertension and hypotension. In view of this, patients who were known to suffer from cardiovascular disease, hypertension or pulmonary disease were not considered for treatment. Patients with a history of allergy and those over the age of 75 years were also excluded.

The trial was carried out over a period of 54 months. All patients randomized into the trial have been followed up to the closing date by the Consultant Surgeon who performed the original operation. The diagnosis of recurrence of disease was based in general on clinical criteria; no CEA estimations were made.

Randomization was carried out according to statistical tables supplied by the Department of Medical Statistics, Oxford University. The statistical analyses of the survival curves has been carried out by drawing life tables and applying the log-rank test (Peto *et al.*, 1977).

*C. parvum* was provided as a generous gift by Dr T. J. Priestman, Wellcome Research Foundation, Beckenham, Kent.

### RESULTS

A total of 44 patients were randomized into the treatment group and 48 into the control group (Table I). The average age of the control patients was rather less than that of the treatment group, being 58·9 years and 63·4 years respectively. The sex distribution between the groups was unequal: 38·6% (17/44) of the treatment group were males compared with 66·6% (32/48) in the control group.

TABLE I.—*Analysis of patients entered into the clinical trial*

| | Treatment | Control |
|---|---|---|
| Total | 44 | 48 |
| Male | 17 | 32 |
| Female | 27 | 16 |
| Mean age (yr) | 63.4 | 58.9 |

Dukes B tumours were evenly distributed between the two groups, but there were more Dukes C lesions in the control group (Table II).

TABLE II.—*Staging of patients in trial*

| Dukes stage | Treatment | Control |
|---|---|---|
| B | 35 | 31 |
| C | 9 | 17 |

It was planned to give 10 infusions of CP, but it became apparent that in many patients it would not be reasonable to pursue this. Although it was anticipated that tolerance to the side effects of CP would increase after the first few infusions, this only occurred in the minority of patients, and in only 7 out of the 44 patients was the full course given. Over half the group, however, received 5 or more infusions. Eight patients randomized to treatment received none. These have been included in the analysis of the figures as treatment patients.

Recurrence of cancer has occurred to date in over 27% of the treatment patients (12/44), and nearly 30% (13/44) of them are dead (Table III, Fig. 1). Three of these deaths have been from causes other than colorectal cancer; two patients died from myocardial infarctions, one during an apparently uneventful convalescence and before receiving any CP;

TABLE III.—*Fate of patients in trial*

| | Treatment | Control |
|---|---|---|
| Deaths | 13 | 12 |
| Recurrences | 12 | 13 |
| Dead due to recurrence | 10 | 11 |
| Dead from other causes | 3 | 1 |

FIG. 1.—Life-table analysis of survival in the two groups. (. . . . . , control. ——, treatment).

the third patient died from a second malignancy which developed in the uterus and was histologically confirmed as unrelated to the colonic cancer.

Nine patients developed recurrent cancer during the course of CP infusions. Seven patients had the full course of 10 infusions, and only one of them has developed recurrence of disease. An almost identical percentage of the control group of patients has developed signs of recurrent cancer. Although the recurrence rate is 27% (13/48) the percentage who are dead is rather lower than in the treatment group, being 25% (12/48). Only one death in the control group was not due to recurrent cancer, this patient dying from acromegalic cardiomyopathy.

The mean intervals before recurrence are virtually identical: 17·3 months in the treatment group and 17 months in the controls.

When the 36 patients who actually received CP are considered, 10 have so far died of recurrent cancer, the median times to recurrence and death being 18·9 months and 28 months respectively. The principal sites of recurrence were intra-abdominal in both groups.

Of the 25 patients receiving 5 or more infusions, 8 (32%) are dead, 7 from recurrent cancer. Nor did those patients receiving prolonged treatment show any difference in the sites of recurrence.

When life-table analysis was applied to the survival figures according to the Dukes stage there was still no difference between the groups (Fig. 2).

Table IV shows the principal sites where tumour recurrence first became evident.

TABLE IV.—*Analysis of recurrences in trial patients*

| Principal sites of recurrence | Treatment | Control |
|---|---|---|
| Local | 2 | 3 |
| Intra-abdominal | 9 | 9 |
| Extra-abdominal | 1 | 1 |

FIG. 2.—Life-table analysis of survival in the two groups according to tumour stage at presentation. (. . . . ., control. ——, treatment).

It does not appear that treatment has influenced the pattern of recurrence. In clinical practice it is often impossible to be sure of the extent of recurrence of cancer, and for the purpose of this trial the most obvious sites of recurrence have been assumed also to be the main sites. Most of the recurrences in both groups have been within the abdominal cavity. In a few cases recurrence appeared to be genuinely localized, for example at the site of the anastomosis or involving structures near the site of the original tumour excision. Two patients in the control group had second resections for locally recurrent cancer, and both are still alive 30 and 24 months from their second operations.

### DISCUSSION

There was no difference in survival or in the incidence of recurrence between the control and treatment groups in this trial of adjuvant immunotherapy in Dukes B and C colorectal cancer. However, both the treatment and the control groups of patients show better survival figures than in the retrospective study on patients presenting with colorectal cancer in Oxford reported by Gill *et al.* (1977) and Gill & Morris (1978). This is probably explained by the selection of younger and relatively healthy patients for inclusion in the trial. The observation also emphasizes the dangers in the use of historical controls.

The Oxford retrospective study also showed that there were no significant sex differences in prognosis after surgery. It might be that the preponderance of male patients in the control group of this study has biased the results, because the overall improvement in prognosis for female patients has long been known (Gabriel, 1948).

The death of a patient who sustained a myocardial infraction within 24 h of treatment led to concern that the trial should be stopped (Gill *et al.*, 1977). This patient had tolerated the first infusion well, but during the second had a marked hypotensive episode from which he appeared to recover satisfactorily. However, 12 h later he developed crushing chest pain and died with irrefutable evidence of an acute myocardial infraction. Although no further tragedies occurred after the adoption of a more rigid exclusion policy, this resulted in very low input to the trial, with the exclusion of a high percentage of otherwise suitable patients because of concern about the side-effects of CP.

At the beginning of this trial it seemed reasonable to aim for a course of infusions. Unfortunately the tolerance which was reported to build up after the first few infusions (Israël, 1975) developed only in a minority of our patients. Indeed, some patients suffered from increasingly severe side-effects. Most of the patients who did not complete the course of treatment were withdrawn by the investigating team, but 2 found the side-effects intolerable and requested that treatment be stopped.

The 8 treatment-group patients who did not receive CP comprised 1 lady who died during convalescence, and 7 who declined treatment having previously given informed consent before randomization. As a full discussion of the side-effects and nature of the proposed treatment took place before randomization, it is perhaps understandable that these patients had second thoughts about undergoing the infusions once they were discharged from hospital. No subsequent attempt was made to persuade these patients to have the treatment.

The poor outlook for Dukes B and C colorectal cancer has prompted many other clinical trials of adjuvant treatment after surgery (Li & Ross, 1976; Valdivieso & Mavligit, 1978). In several of these, chemotherapy with 5-fluorouracil (FU) has been used, and in other trials chemotherapy was combined with attempted stimulation of the immune system (MacDonald, 1976). The most commonly adopted means of immune stimulation has been repeated doses of BCG, usually administered by scarification. As this treatment was often very unpleasant, painful and sometimes caused local ulceration, fractions of the BCG cell wall have also been used, since these were thought to be less likely to cause side-effects. The results from most of these trials are inconclusive or not yet published. Where claims of benefit have been made (*e.g.* the M. D. Anderson group), the structure of the trial must be questioned (Valdivieso & Mavligit, 1978). In that trial patients were treated after surgery for Dukes C cancers in two ways. Some patients received repeated scarification with BCG alone, while others had the same treatment combined with oral FU. BCG treatment involved scarification weekly for 3 months, and then on alternate weeks. FU treatment involved 150 mg/m$^2$ of the drug taken orally 4 times a day for 5 days, once every 4 weeks over 2 years. Each group had a prolonged disease-free interval, and a survival curve that matched that of a historical control group of patients who had suffered from Dukes B cancer. Since both treatment groups had similar disease-free intervals and survival times it seemed resaonable to attribute this to the BCG rather than the FU. However, these figures must be criticized because of the use of a historical control. The apparent improvement in the outcome of treatment for Dukes C patients could well have been due to other factors, for example a change in the type of patient presenting for surgery. If the survival of the control group with Dukes C lesions is carefully considered in isolation, it appears particularly poor and hence the improvement in prognosis for both treatment groups in this study may not be real.

Israël's report (1975) of the beneficial effect of systemic CP in advanced human malignancy engendered considerable enthusiasm for this approach. However, confirmation of a beneficial effect of CP in advanced cancer has not been provided from other centres. (Fisher *et al.*, 1976; Chare *et al.*, 1978).

The ability of a treatment to influence the growth and spread of tumours in laboratory animals supports the investigation of its use in human malignancy. But laboratory results are not necessarily of clinical relevance. Even in laboratory animals, systemic CP has a weak action against large tumour masses, and is more effective in suppressing the development of metastases (Sadler & Castro, 1976) CP in our trial has not influenced the recurrence of disease.

One objection to the use of immunostimulants is the lack of specificity for target cells, and the risk of suppressing existing *specific* defence mechanisms, *e.g.*, increasing a population of suppressor T cells, leading to enhanced tumour growth. None of the early human studies with CP in advanced human cancers suggested that this did happen, but there was a clear need for caution in monitoring the progress of the patients in our trial (Fisher, 1978; James *et al.*, 1978).

The trial has now been closed. No obvious beneficial or deleterious effect of CP infusions was found in Dukes B or C colorectal cancers, as determined by patient survival, cancer recurrence and the time of recurrence. However, the small numbers studied do not allow us to exclude the possibility of there being a small effect, beneficial or deleterious, which would only be apparent with many more patients.

This trial received financial assistance from the Cancer Research Campaign.

We wish to acknowledge our gratitude to the following Consultants for allowing their patients to enter the trial:

*The John Radcliffe Hospital, Oxford.*—B. J. Britton, N. E. Dudley, M. H. Gough, M. G. W. Kettlewell, E. Lee, P. J. Morris, H. W. Steer, D. J. Tibbs, C. Webster.

*King Edward VII Hospital, Windsor.*—D. W. Bain, R. J. Luck.

*Stoke Mandeville Hospital, Aylesbury.*—C. J. Smallwood

### REFERENCES

Bull, D. M., Leibach, J. R., Williams, M. A. & Helms, R. A. (1973) Immunity to colon cancer assessed by antigen induced inhibition of mixed mononuclear cell migration. *Science.*, **181**, 957.

Carter, S. K. (1976) Current status of immunotherapy for large bowel cancer. *Cancer, Immunol. Immunother.*, **1**, 199.

Chare, M. J. B., Webster, D. J. T. & Baum, M. (1978) Clinical experience in the use of *C. parvum* in the treatment of locally advanced carcinoma of the breast. *Devel. Biol. Stand.*, **38**, 495.

FISHER, R. A. (1978) *In vitro* and *in vivo* effects of *Coryhebacterium parvum on* lymphocyte transformation. *Devel. Biol. Stand.*, **38**, 461.

FISHER, B., RUBEN, H., SARTIANO, G. ENNIS, L. & WOODMARK, W. (1976) Observations following *Corynebacterium parvum* administration to patients with advanced malignancy. *Cancer.*, **38**, 119.

GABRIEL, W. B. (1948) *Principles and Practice of Rectal Surgery.* (4th. Ed.) London: H. K. Lewis.

GILL, P. G. & MORRIS, P. J. (1978) The survival of patients with colorectal cancer treated in a regional hospital. *Br. J. Surg*, **65**, 17.

GILL, P. G., MORRIS, P. J. & KETTLEWELL, M. (1977) The complications of intravenous *Corynebacterium parvum* infusion. *Clin. Exp. Immunol.*, **30**, 229.

GOLD, P. & FREEDMAN, S. O. (1965) Demonstration of tumour-specific antigens on human colonic carcinomata by immunological tolerance and absorption techniques. *J. Exp. Med.*, **121**, 439.

HOLLINSHEAD, A. C., MCWRIGHT, C. G., ALFORD, T. C. & GLEW, D. H. (1972) Separation of skin reactive intestinal cancer antigen from the carcinoembryonic antigen of gold. *Science*, **177**, 887.

ISRAËL, L. (1975) Report on 414 cases of human tumours treated with *Corynebacterium parvum.* In Corynebacterium parvum: *Applications in Experimental and Clinical Oncology.* (Ed. Halpern). New York: Plenum Press p. 389.

JAMES, K., MERRIMAN, J., WOODRUFF, M. F. A. (1978) Further studies on the serological effects of *C. parvum* immunotherapy in cancer patients. *Devel. Biol. Stand.*, **38**, 501.

LI, M. C. & ROSS, S. T. (1976) Chemoprophylaxis for patients with colorectal cancer. Prospective study with 5-year follow up. *Jama*, **245**, 2825.

LOCKHART-MUMMERY, H. E., RITCHIE, J. K. & HAWLEY, P. R. (1976) The results of surgical treatment for carcinoma of the rectum at St Mark's Hospital from 1948 to 1972. *Br. J. Surg.*, **63**, 673.

MACDONALD, J. S. (1976) The immunobiology of colorectal cancer. *Semin. Oncol.*, **3**, 421.

NAIRN, R. C., NIND, A. D. D., GULI, E. P. G. & 4 others (1971) Immunological reactivity in patients with carcinoma of colon. *Br. Med. J.*, iv, 706.

OFFICE OF POPULATION CENSUSES AND SURVEYS (1977) Mortality Statistics for England and Wales.

PETO, R., PIKE, M. C., ARMITAGE, D. & 7 others (1977) Design and analysis of randomized clinical trials requiring prolonged observation of each patient: Analysis and examples. *Br. J. Cancer*, **35**, 1.

PIHL, E., NAIRN, R. C., NIND, A. P. & 4 others (1976) Correlation of regional lymph node *in vitro* anti-tumour immunoreactivity histology with colorectal carcinoma. *Cancer Res.*, **36**, 3665.

SADLER, T. E. & CASTRO, J. E. (1976) The effects of *Corynebacterium parvum* and surgery on the Lewis lung carcinoma and its metastases. *Br. J. Surgery*, **63**, 292.

SCOTT, M. T. (1974*a*) *Corynebacterium parvum* as an immunotherapeutic anti-cancer agent. *Semin. Oncol.*, **1**, 367.

SCOTT, M. T. (1974*b*) *Corynebacterium parvum* as a therapeutic anti-cancer agent in mice. Systemic effects from intravenous injection. *J. Natl Cancer Inst.*, **53**, 855.

SLANEY, G. (1971) Results of treatment of carcinoma in the colon and rectum. In *Modern Trends in Surgery.*, **3**, (Ed. Irving). London: Butterworths. p. 69.

VALDIVIESO, M. & MAVLIGIT, M. G. (1978) Chemotherapy and chemoimmunotherapy of colorectal cancer. *Surg. Clin. North Am.*, **58**, 619.

VOSE, B. M., GALLAGHER, P., MOORE, M. & SCHOFIELD, P. F. (1981) Specific and nonspecific lymphocyte cytotoxicity in colon carcinoma *Br. J. Cancer.*, **44**, 846.

WALKER, R. M. (1972) *Cancer in South West England: Supplementary Report.* Bristol: South Western Regional Cancer Bureau.

WANEBO, H. J., RAO, B., ATTIYEH, F., PINSKY, C., MIDDLEMAN, P. & STEARNS, M. Immune reactivity in patients with colorectal cancer: Assessment of biologic risk by immunoparameters. *Cancer*, **45**, 1254.

WHITTAKER, M. & GOLIGHER, J. C. (1976) The prognosis after surgical treatment for carcinoma of the rectum. *Br. J. Surg.*, **63**, 384.


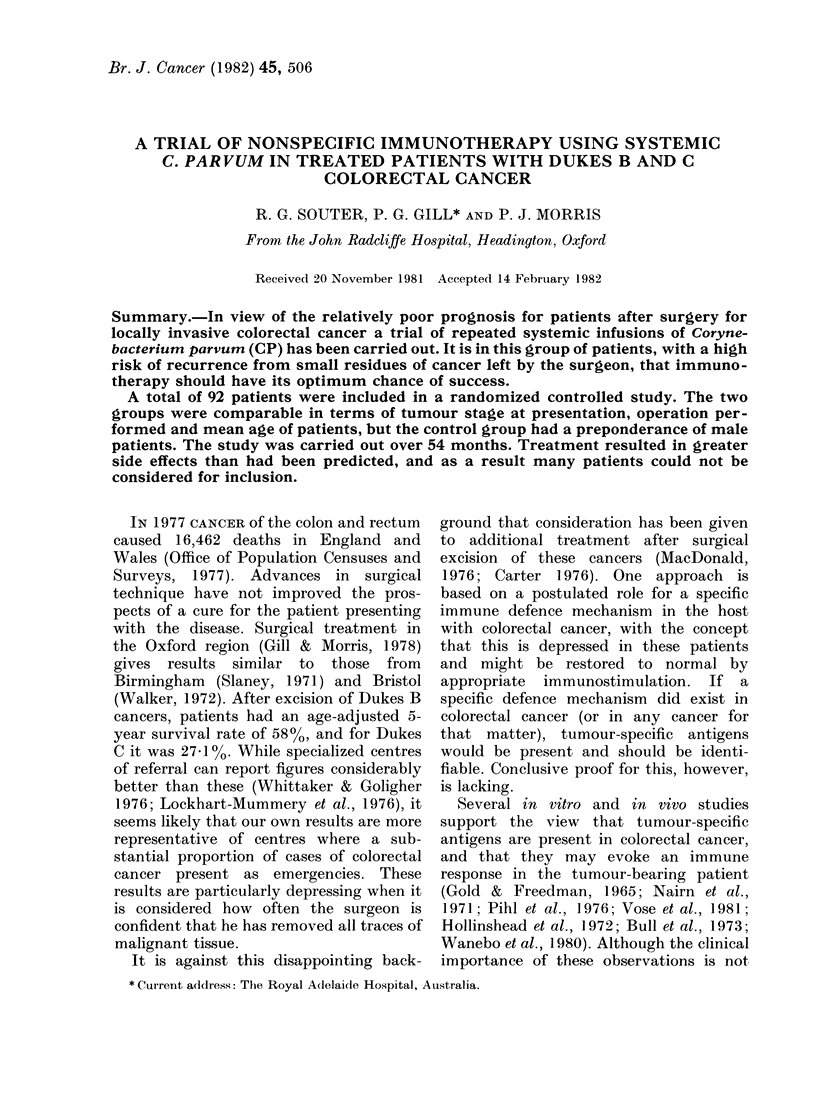

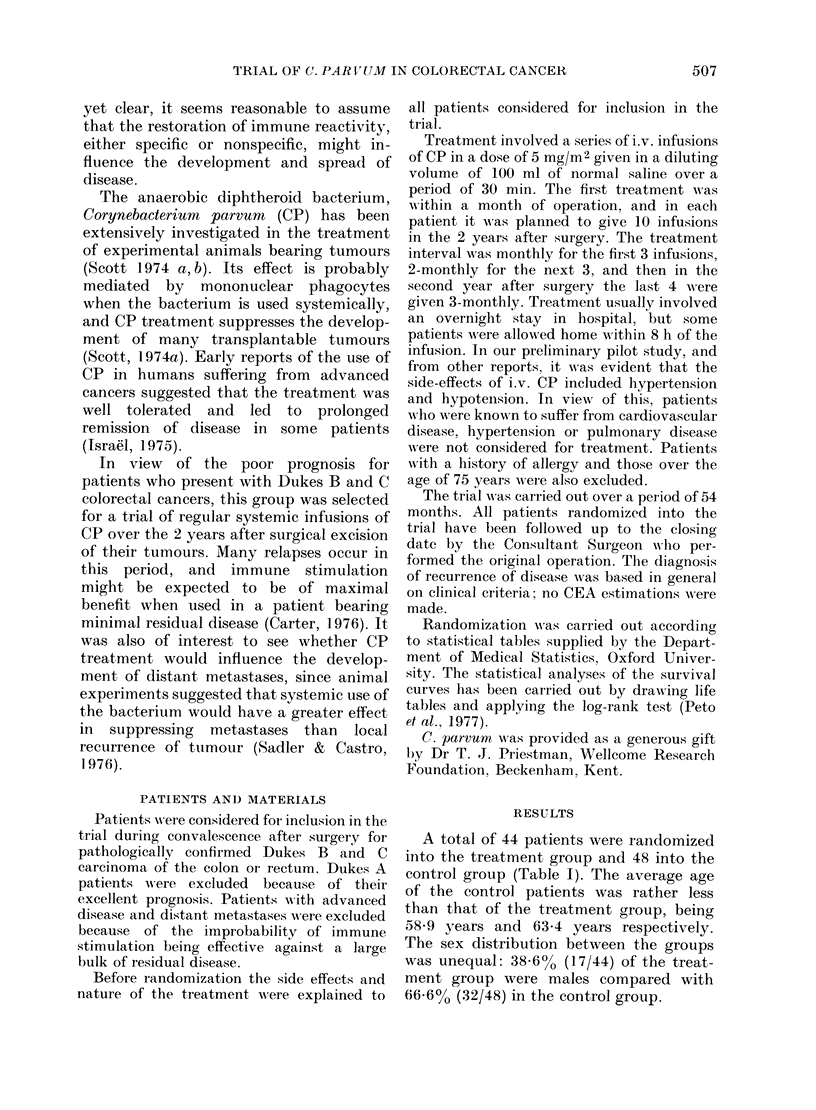

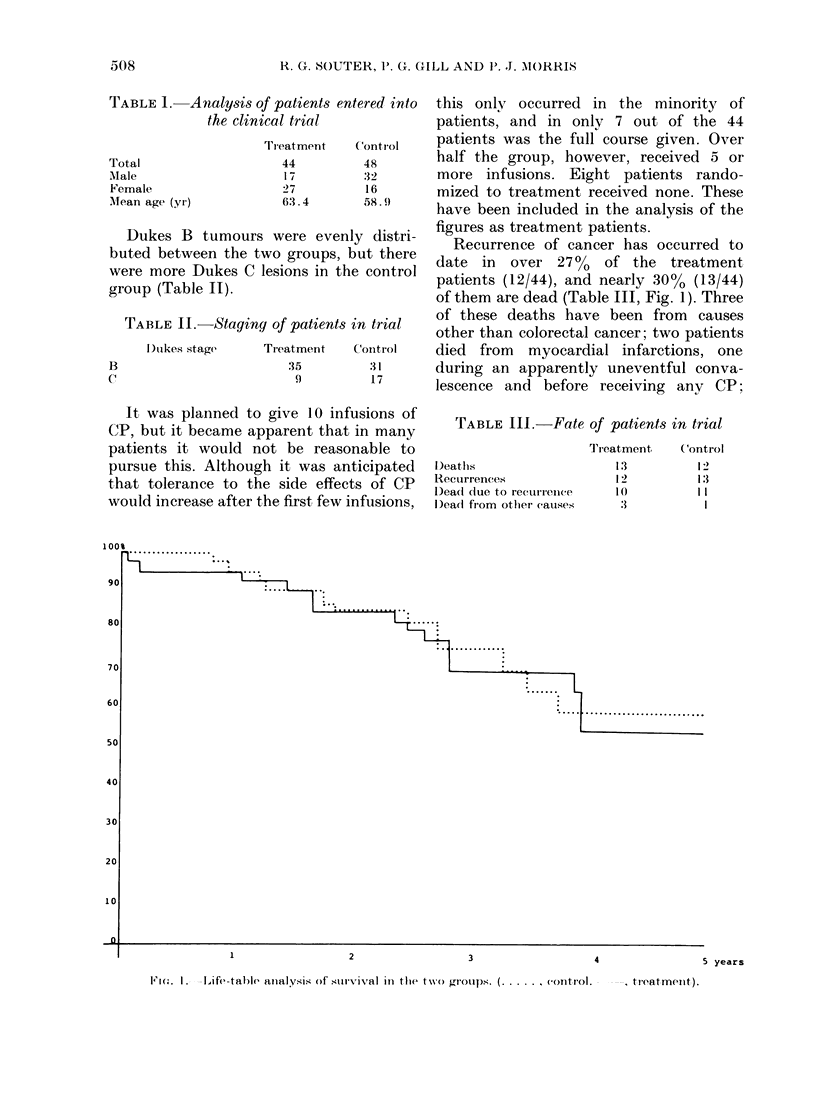

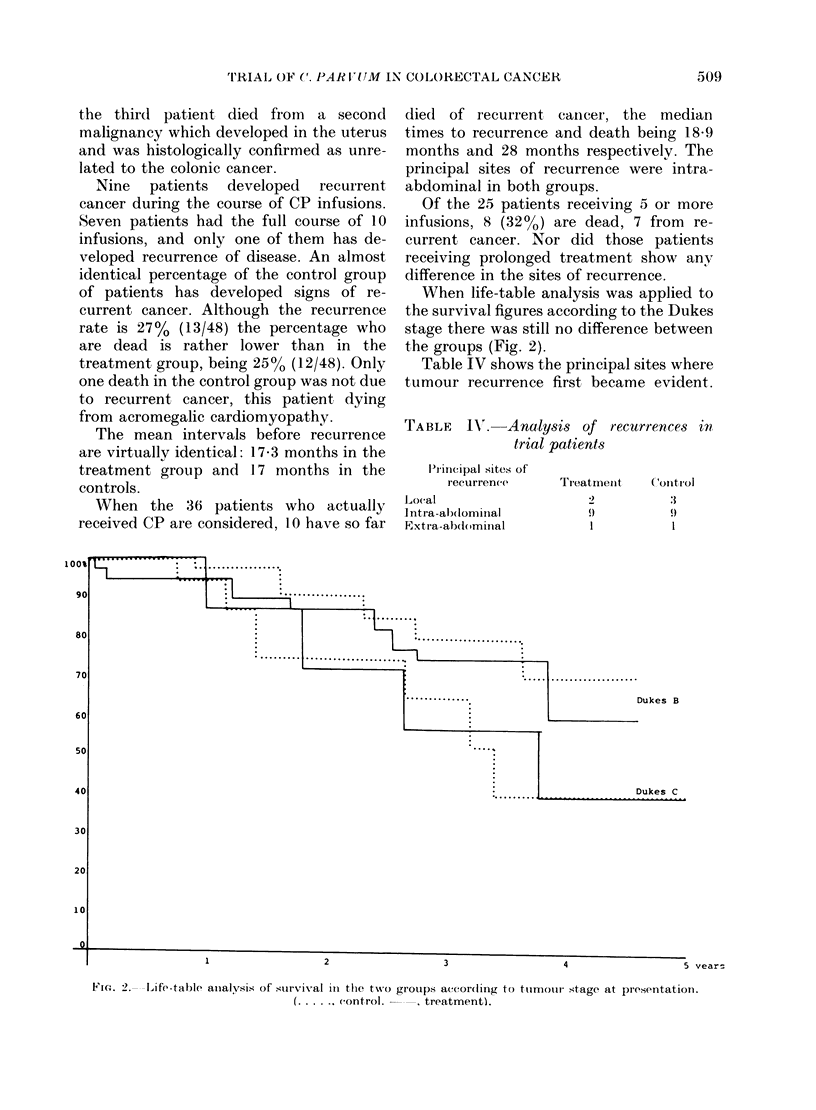

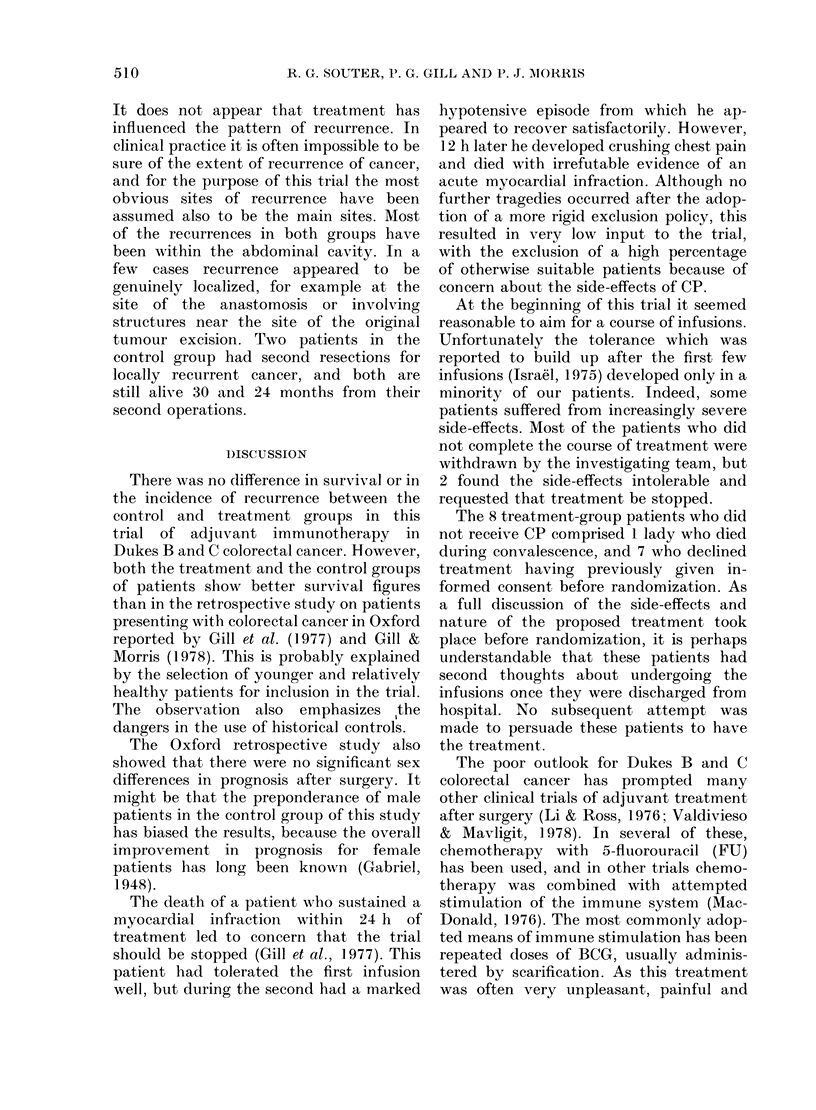

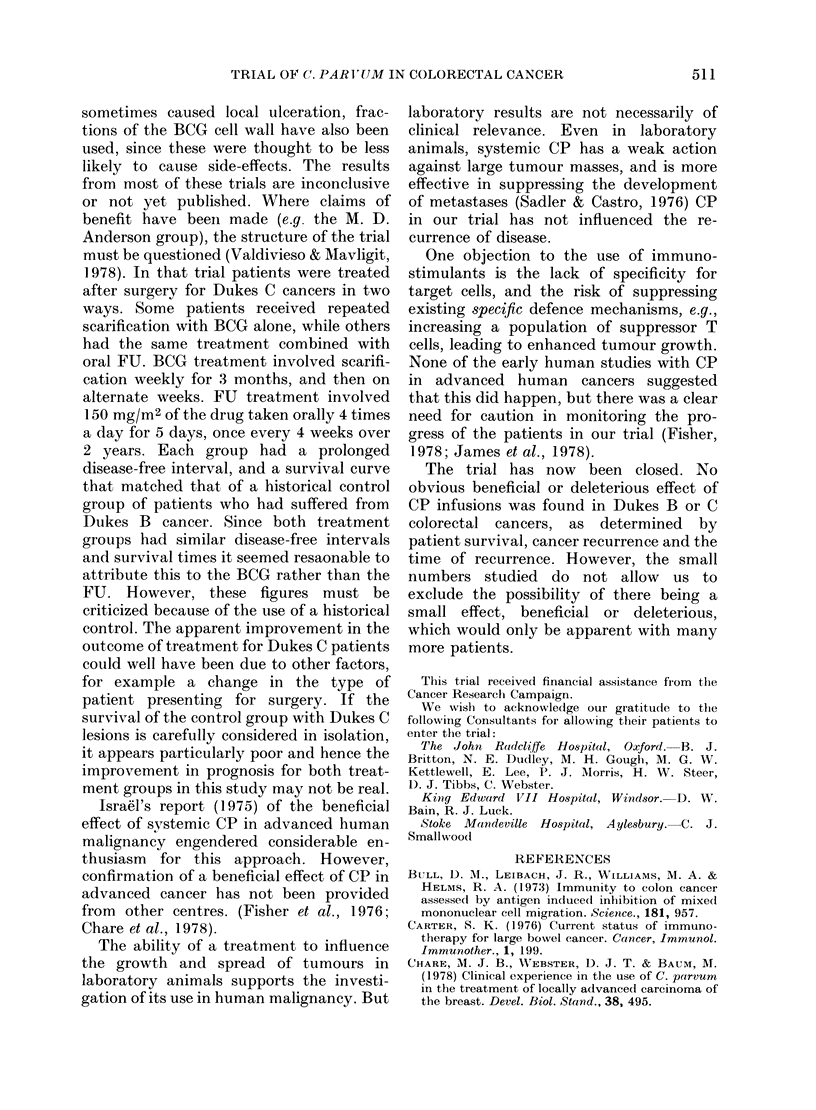

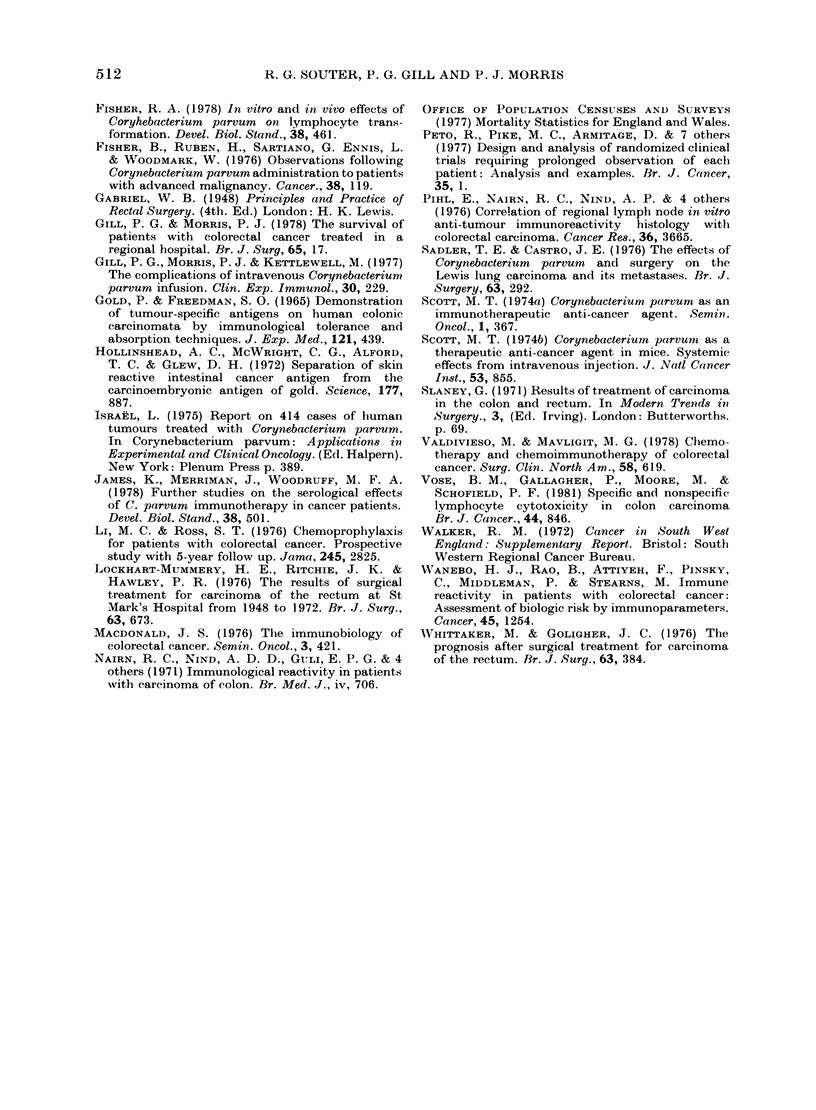


## References

[OCR_00585] Bull D. M., Leibach J. R., Williams M. A., Helms R. A. (1973). Immunity to colon cancer assessed by antigen-induced inhibition of mixed mononuclear cell migration.. Science.

[OCR_00596] Chare M. J., Webster D. J., Baum M. (1977). Clinical experience in the use of C. parvum in the treatment of locally advanced carcinoma of the breast.. Dev Biol Stand.

[OCR_00609] Fisher B., Rubin H., Sartiano G., Ennis L., Wolmark N. (1976). Observations following Corynebacterium parvum administration to patients with advanced malignancy. a phase I study.. Cancer.

[OCR_00604] Fisher R. A. (1977). In vitro and in vivo effects of Corynebacterium parvum on lymphocyte transformation.. Dev Biol Stand.

[OCR_00629] GOLD P., FREEDMAN S. O. (1965). DEMONSTRATION OF TUMOR-SPECIFIC ANTIGENS IN HUMAN COLONIC CARCINOMATA BY IMMUNOLOGICAL TOLERANCE AND ABSORPTION TECHNIQUES.. J Exp Med.

[OCR_00624] Gill P. G., Morris P. J., Kettlewell M. (1977). The complications of intravenous Corynebacterium parvum infusion.. Clin Exp Immunol.

[OCR_00619] Gill P. G., Morris P. J. (1978). The survival of patients with colorectal cancer treated in a regional hospital.. Br J Surg.

[OCR_00635] Hollinshead A. C., McWright C. G., Alford TC GLEW D. H., Gold P., Herbeman R. B. (1972). Separation of skin reactive intestinal cancer antigen from the carcinoembryonic antigen of Gold.. Science.

[OCR_00649] James K., Merriman J., Woodruff M. F., McCormick J. N., McBride W. H., Innes J., Horne N. W. (1977). Further studies on the serological effects of C. parvum immunotherapy in cancer patients.. Dev Biol Stand.

[OCR_00655] Li M. C., Ross S. T. (1976). Chemoprophylaxis for patients with colorectal cancer. Prospective study with five-year follow-up.. JAMA.

[OCR_00660] Lockhart-Mummery H. E., Ritchie J. K., Hawley P. R. (1976). The results of surgical treatment for carcinoma of the rectum of St Mark's Hospital from 1948 to 1972.. Br J Surg.

[OCR_00667] Mcdonald J. S. (1976). The immunobiology of colorectal cancer.. Semin Oncol.

[OCR_00673] Nairn R. C., Nind A. P., Guli E. P., Davies D. J., Rolland J. M., McGiven A. R., Hughes E. S. (1971). Immunological reactivity in patients with carcinoma of colon.. Br Med J.

[OCR_00686] Pihl E., Nairn R. C., Nind A. P., Muller H. K., Hughes E. S., Cuthbertson A. M., Rollo A. J. (1976). Correlation of regional lymph node in vitro antitumor immunoreactivity histology with colorectal carcinoma.. Cancer Res.

[OCR_00692] Sadler T. E., Castro J. E. (1976). The effects of Corynebacterium parvum and surgery on the Lewis lung carcinoma and its metastases.. Br J Surg.

[OCR_00698] Scott M. T. (1974). Corynebacterium parvum as an immunotherapeutic anticancer agent.. Semin Oncol.

[OCR_00715] Valdivieso M., Mavligit G. M. (1978). Chemotherapy and chemoimmunotherapy of colorectal cancer. Role of the carcinoembryonic antigen.. Surg Clin North Am.

[OCR_00720] Vose B. M., Gallagher P., Moore M., Schofield P. F. (1981). Specific and non-specific lymphocyte cytotoxicity in colon carcinoma.. Br J Cancer.

[OCR_00738] Whittaker M., Goligher J. C. (1976). The prognosis after surgical treatment for carcinoma of the rectum.. Br J Surg.

